# In Memoriam—Dr. William S. Armstrong

**Published:** 1896-03

**Authors:** James B. Baird, W. S. Kendrick

**Affiliations:** Committee. Atlanta Society of Medicine. Atlanta; Committee. Atlanta Society of Medicine. Atlanta


Committee.

---

### IN MEMORIAM—DR. WILLIAM S. ARMSTRONG.

ATLANTA SOCIETY OF MEDICINE.
ATLANTA, February 18, 1896.

The undersigned committee, by appointment of the President, respectfully submits the following report:

Dr. Armstrong died suddenly, of organic disease of the heart, Tuesday evening, February 11, 1896, in the reception room of the Grady Hospital, at which place he had arrived only a few minutes before for the purpose of attending a regular meeting of the medical board, to the presidency of which he was recently elected. Dr. Armstrong was born in Washington, Wilkes county, Georgia, in 1838. After a liberal preliminary education he commenced the study of medicine under Dr. W. H. Lane, of Washington, and after one course of lectures in Augusta he matriculated in the medical department of the University of New York, and graduated from that institution in 1859. He served with credit to himself as surgeon in the Confederate army from 1861 to 1865. Soon after the close of the war he went to Europe to further prosecute his medical studies, and upon his return home he located in Atlanta and commenced the general practice of medicine. In 1867 he was elected to the chair of Anatomy in the Atlanta Medical College, after having served one year as demonstrator, and he continued to fill that position with distinguished ability to the day of his death. In 1890 Clinical Surgery was added to the chair of Anatomy. He was one of the original members of the surgical staff of the Grady Hospital, and proved in that capacity an ardent and invaluable worker. He was a member and the president of the board of health of the city of Atlanta for fourteen years, until he resigned in 1893, and accomplished extremely valuable results for the sanitary interests of the city during a very important period of its history and growth.

In the practice of his profession he exhibited a strong predilection for surgery. His enthuasism in his work was remarkable and contagious. His earnestness and fidelity was conspicuous and invariable.

During the latter years of his life he performed many of the most intricate and difficult of the major surgical operations, and achieved signal and brilliant results.

His excellence of character, his unselfishness, and his uniform kindness and consideration were universally recognized, and with all ot his admirable traits and all of his professional accomplishments he was as modest and unassuming as a child.

His whole life—public, private, religious, and professional—was free from stain. It was marked by faithfulness, honesty, and truth, and should serve as a shining example to those who desire to meet their obligations to their Master and their fellow-men.

JAMES B. BAIRD, M.D.,
W. S. KENDRICK, M.D.,
Committee.

---

RESOLUTIONS BY MEDICAL STAFF OF GRADY HOSPITAL.

ATLANTA, February 14, 1896.

WHEREAS, In the supreme wisdom of Almighty God, our friend, our brother, and our colleague, William Simpson Armstrong, M.D., has been stricken by the hand of death; and

WHEREAS, The summons from life to the realms of the great hereafter came in a moment, in the twinkling of an eye, and he was snatched, without warning, from the circle of his associates and his comrades, while in the discharge of his professional and official duties, without a pain or a pang to mark the transition from the scenes of his labors and of his triumphs to the regions eternal; therefore be it

*Resolved*, That in solemn contemplation of the inestimable loss to his family, his profession, and his country, we record our recognition of his worth and of his unwavering fidelity to every obligation, to every truth, and in every relation of life. Yet, in humble submission to the edict of omniscience, with bowed heads and heavy hearts, we yield, unmurmuring, to the divine decree.

*Resolved*, That in the estimation of those who knew him best he was a christian without hypocrisy, a friend without deceit, a citizen without reproach, a physician without pretense, a man without guile, who, in his active but unostentatious life, illustrated all that is noblest and best in humanity.

*Resolved*, That these resolutions be furnished the daily papers and the local medical journals for publication, and that our profound sympathies be extended to the family of our brother.

W. S. KENDRICK,
JAMES B. BAIRD,
H. P. COOPER,
Committee.

---

### RESOLUTIONS BY THE FACULTY OF THE ATLANTA MEDICAL COLLEGE.

ATLANTA, February 14, 1896.

WHEREAS, The subject of these resolutions, William Simpson Armstrong, M.D., by the decree of an all-wise providence, died suddenly while presiding at a meeting of the medical staff of the Grady Hospital, February 11, 1896; therefore be it

*Resolved* 1. That in the death of our beloved and highly esteemed professional brother, co-worker, and friend, the Atlanta Medical College has not only lost one of its strongest supporters, but a most zealous, active, and competent member of its faculty, who discharged every duty devolving on him with all the earnestness and capability that characterized his noble and busy life. Engaged each day with the arduous duties of professiolal work, he never failed to meet promptly every demand made upon him by the institution he loved so well. Plain and simple in manners, unobtrusive and entirely without ostentation, his early medical training and continued study to the end of his career, joined to his fine practical sense, entitled him to the highest place among his fellows, to the unbounded confidence of those to whom he ministered, and to the rich harvest of professional honors he yearly reaped. As a teacher of anatomy and clinical surgery he became distinguished, and throughout all this country stood without a peer. For many years he was a general surgeon of prominence, but some of his recent operations have rendered his name illustrious. Professionally honest, upright, consistent, and a manly man, he was born a gentleman of the old school. Despising the small and unclean things in professional life, he goes down to his grave with a record unblemished, with a name untarnished, and with a life whose ending, touching, sudden, and tragic, has been filled with deeds of love and acts of charity. He died under the very shadow of the institution which he loved so well, and which was a part of the crowning glory of his earthly career. It matters not to the living whether his life-work be recorded on the pages of history, or whether a marble shaft marks the place where he sleeps, for he will still live in the sweet memories of those to whom he so kindly ministered. At his post of duty he died, surrounded by some of his most intimate medical friends. The faculty of the Atlanta Medical College will miss his coming and going, but ripe for the sickle of death, full of years and honors, he has been gathered to his fathers.

*Resolved* 2. That these resolutions be spread upon the pages of our minute book, and a copy be furnished the family of the deceased, with the tenderest sympathies of this faculty.

